# A novel approach of chemical mechanical polishing using environment-friendly slurry for mercury cadmium telluride semiconductors

**DOI:** 10.1038/srep22466

**Published:** 2016-03-01

**Authors:** Zhenyu Zhang, Bo Wang, Ping Zhou, Dongming Guo, Renke Kang, Bi Zhang

**Affiliations:** 1 Key Laboratory for Precision and Non-Traditional Machining Technology, Ministry of Education, Dalian University of Technology, Dalian 116024, China; 2 Changzhou Institute of Dalian University of Technology, Changzhou 213164, China; 3 Department of Mechanical Engineering, University of Connecticut, Storrs, CT 06269, USA

## Abstract

A novel approach of chemical mechanical polishing (CMP) is developed for mercury cadmium telluride (HgCdTe or MCT) semiconductors. Firstly, fixed-abrasive lapping is used to machine the MCT wafers, and the lapping solution is deionized water. Secondly, the MCT wafers are polished using the developed CMP slurry. The CMP slurry consists of mainly SiO_2_ nanospheres, H_2_O_2_, and malic and citric acids, which are different from previous CMP slurries, in which corrosive and toxic chemical reagents are usually employed. Finally, the polished MCT wafers are cleaned and dried by deionized water and compressed air, respectively. The novel approach of CMP is environment-friendly. Surface roughness R_a_, and peak-to-valley (PV) values of 0.45, and 4.74 nm are achieved, respectively on MCT wafers after CMP. The first and second passivating processes are observed in electrochemical measurements on MCT wafers. The fundamental mechanisms of CMP are proposed according to the X-ray photoelectron spectroscopy (XPS) and electrochemical measurements. Malic and citric acids dominate the first passivating process, and the CMP slurry governs the second process. Te^4+^3d peaks are absent after CMP induced by the developed CMP slurry, indicating the removing of oxidized films on MCT wafers, which is difficult to achieve using single H_2_O_2_ and malic and citric acids solutions.

Mercury cadmium telluride (Hg_1−x_Cd_x_Te or MCT) has become the most versatile semiconductor for infrared (IR) detectors, in terms of their tunable band gaps over the entire IR range^1^,^2^,^3^,^4^,^5^,^6^,^7^. At present, MCT is the most widely used variable gap semiconductor for IR photodetectors^4^. In addition, MCT is nearly ideal for IR detector system, remaining the leading semiconductor in IR detectors. Nevertheless, the Hg-Te bonds in MCT are extremely weak, resulting in its soft and brittle nature^4^,^8^,^9^. This makes MCT become a difficult-to-machine material. For instance, the hardness of MCT varies from 0.5 to 0.66 GPa^9^,^10^, which is approximately one twenties that of silicon (Si) (12-14 GPa)^11^. The fracture toughness of MCT is 0.158 MPa.m^0.5^, which is about one sixth that of Si (0.9–1.1 MPa.m^1/2^)^8^,^9^. Surface roughness plays an important role to the IR performance of MCT detectors, and therefore the surface roughness of MCT wafers demands less than 1 nm, despite of their soft and brittle nature. This is a stringent requirement for MCT wafers, prior to becoming a high performance IR detector. The conventional machining consists of successive processes for MCT wafers^12^,^13^,^14^,^15^. An MCT wafer is usually lapped using free abrasives with sizes at several microns^12^, and then chemically polished by abrasives with sizes at sub-micron, finally followed by chemical etching using bromine methanol solution^13^,^14^,^15^. These processes have kept basically constant since 2000 year. Nonetheless, bromine and methanol are toxic to both the operators and environment. Scratches are obvious even after the final mechanical polishing by abrasives with size of 50 nm^15^. Furthermore, chemical etching using bromine methanol renders the richness of tellurium element on the MCT surfaces^13^,^15^. Surface roughness root mean square (rms) of 1 nm is reported after chemical etching by bromine methanol^15^. However, the rms is measured by atomic force microscopy (AFM) with a scanning area of 1 × 1 μm^2^. With increasing a measurement area, surface roughness rms increases usually to several nanometers. Few machining process is reported on MCT wafers, as well as related surface science, except for their conventional machining processes. Hence, it is surprising that a novel approach of chemical mechanical polishing (CMP) and its surface science remain virgin ground for MCT wafers, which dominate the performance of IR detectors in a large body of work. No need to reticence, it is a challenge to develop a novel approach of CMP using environment-friendly slurry for MCT semiconductors.

In this study, a novel approach of CMP is developed using environment-friendly slurry for MCT semiconductors. The fundamental mechanisms of CMP are investigated by X-ray photoelectron microscopy (XPS) and electrochemical measurements.

## Results

**Figure 1:**
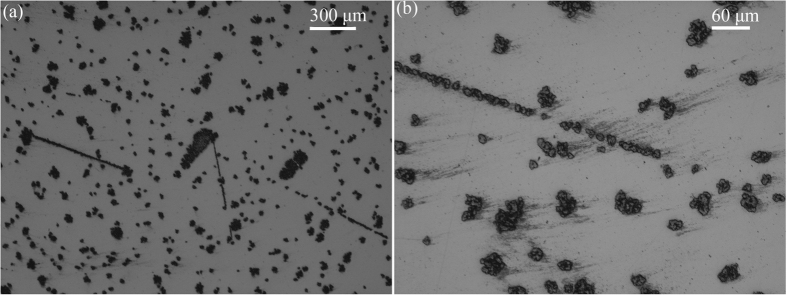
Optical images on an MCT wafer after free abrasive polishing.

Figure 1 shows the optical images on an MCT wafer after free abrasive polishing. Scratches and embedded abrasives are observed on the MCT wafer. Embedded abrasives of alumina are full of on the polished surface, even after flushing of deionized water and dried by compressed air.

**Figure 2:**
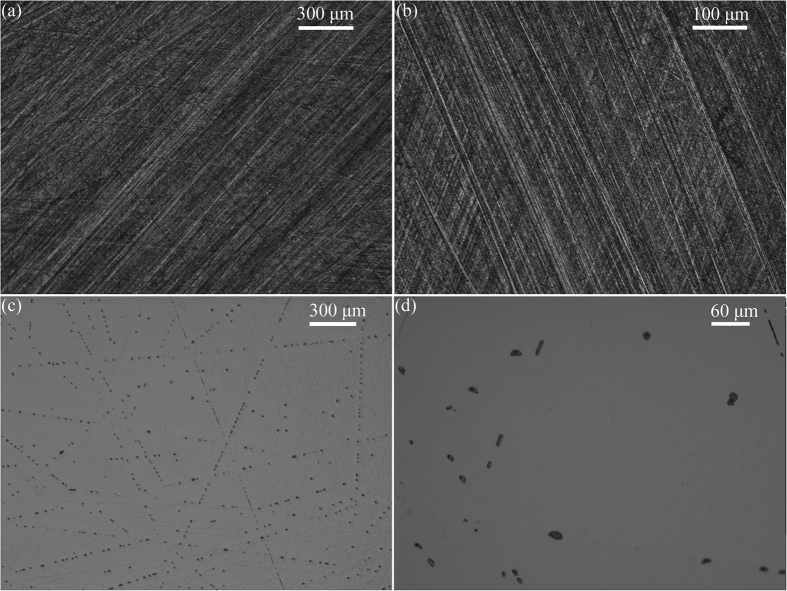
Optical images of surface morphologies after fixed-abrasive lapping (**a**,**b**) by alumina with a size of 5 μm, and CMP (**c**,**d**) using slurry consisting of SiO_2_ and H_2_O_2_ with a volume ratio of 5:1.

Figure 2 shows the optical images of surface morphologies after fixed-abrasive lapping by alumina with a size of 5 μm, and CMP using slurry consisting of SiO_2_ and H_2_O_2_ with a volume ratio of 5:1. There are no embedded abrasives and cracks on the lapped surface of MCT wafers, except for uniform and subtle scratches, as shown in Fig. 2(a,b). The polished surface has shallow scratches, as observed in Fig. 2(c,d), even after CMP using slurry consisting of SiO_2_ and H_2_O_2_ with a volume ratio of 5:1. This is attributed to the absence of malic and citric acids and deviation of H_2_O_2_ from the optimal volume ratio of SiO_2_ to H_2_O_2_ for 5:2.

**Figure 3:**
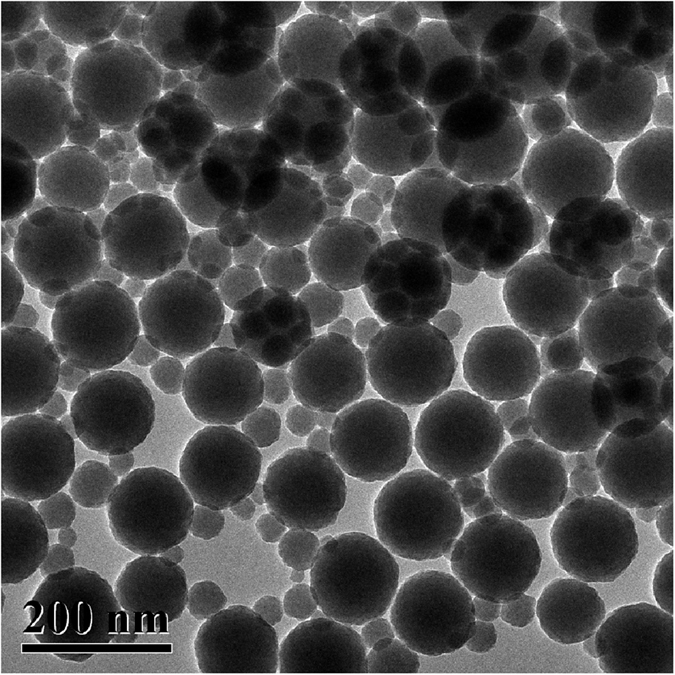
TEM image of SiO_2_ nanospheres in SiO_2_ slurry after evaporation of solution.

Figure 3 shows the TEM image of SiO_2_ nanospheres in SiO_2_ slurry after evaporation of solution. SiO_2_ nanospheres have diameters varying from 20 to 120 nm, and mainly distribute at four diameters of 20, 40, 60, 120 nm.

**Figure 4:**
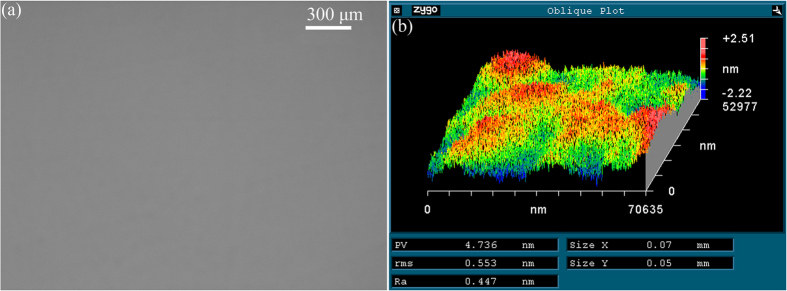
Optical image (**a**) and surface roughness (**b**) on MCT wafers after CMP using environment-friendly CMP slurry with optimal volume ratio.

Figure 4 shows the optical image and surface roughness on MCT wafers after CMP using environment-friendly CMP slurry with optimal volume ratio. The polished surface looks like a mirror, neither scratches, embedded abrasives, nor cracks on the MCT wafers, as illustrated in Fig. 4(a). Surface roughness arithmetic average R_a_, rms, and peak-to-valley (PV) values are 0.447, 0.553, and 4.736 nm, respectively with a measurement area of 50 × 70 μm^2^ on an MCT wafer after CMP using environment-friendly CMP slurry, as seen in Fig. 4(b). The ultralow surface roughness verifies the validity of developed novel approach of CMP, using environment-friendly CMP slurry for such a large measurement area.

**Figure 5:**
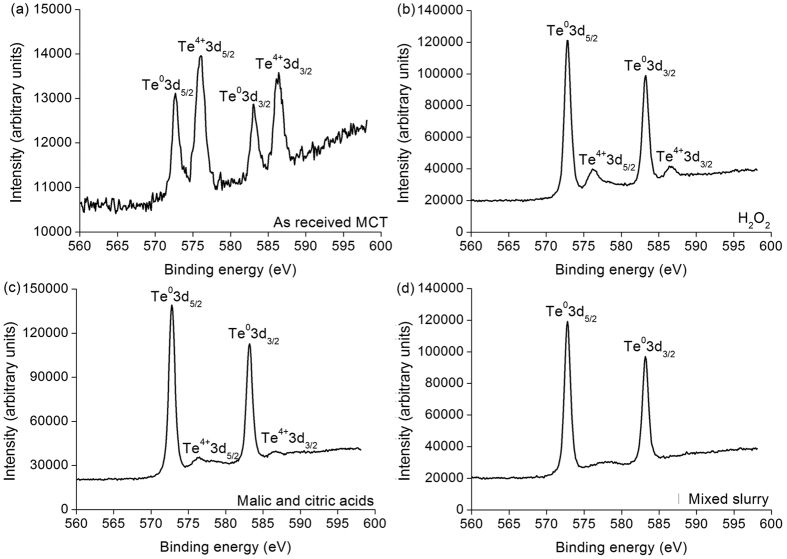
XPS spectra of Te elemental valence states on (**a**) as-received, and (**b**) H_2_O_2_, (**c**) malic and citric acids, and (**d**) mixed slurry polished MCT wafers.

Figure 5 reveals the X-ray photoelectron spectroscopy (XPS) spectra of Te elemental valence states on as-received, and H_2_O_2_, malic and citric acids and mixed slurry polished MCT wafers. Both Te^0^3d and Te^4+^3d peaks are present on an as-received MCT wafer, whereas only Te^0^3d peaks are observed on an MCT wafer after CMP using mixed slurry consisting of H_2_O_2_, SiO_2_, and malic and citric acids^13^,^16^,^17^. Te^4+^3d peaks are very weak on MCT wafers after polishing respectively by H_2_O_2_ and malic and citric acids. Moreover, the Te^4+^3d peaks on an MCT wafer polished by malic and citric acids are weaker than those by H_2_O_2_.

**Figure 6:**
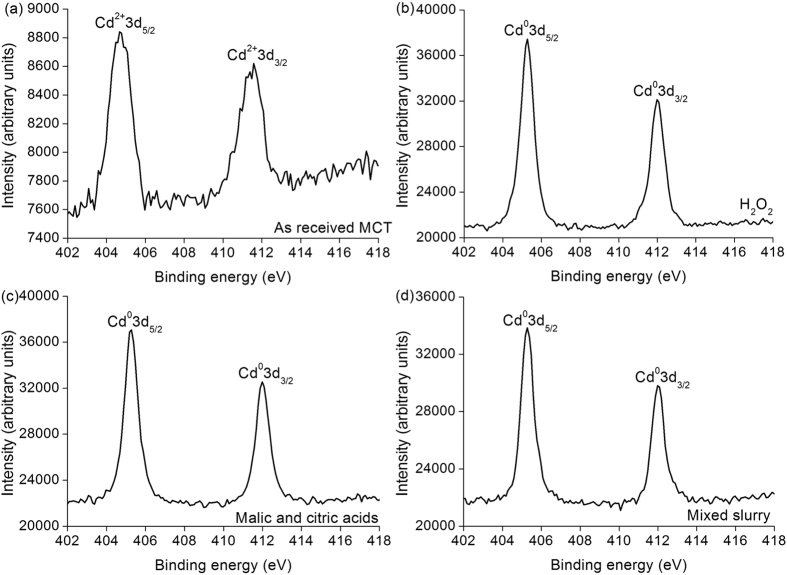
XPS spectra of Cd elemental valence states on (**a**) as-received, and (**b**) H_2_O_2_, (**c**) malic and citric acids, and (**d**) mixed slurry polished MCT wafers.

Figure 6 illustrates the XPS spectra of Cd elemental valence states on as-received, and H_2_O_2_, malic and citric acids, and mixed slurry polished MCT wafers. Cd^0^3d peaks appear on all the polished MCT wafers, except the Cd^2+^3d peaks on the as-received MCT wafers^18^,^19^.

**Figure 7:**
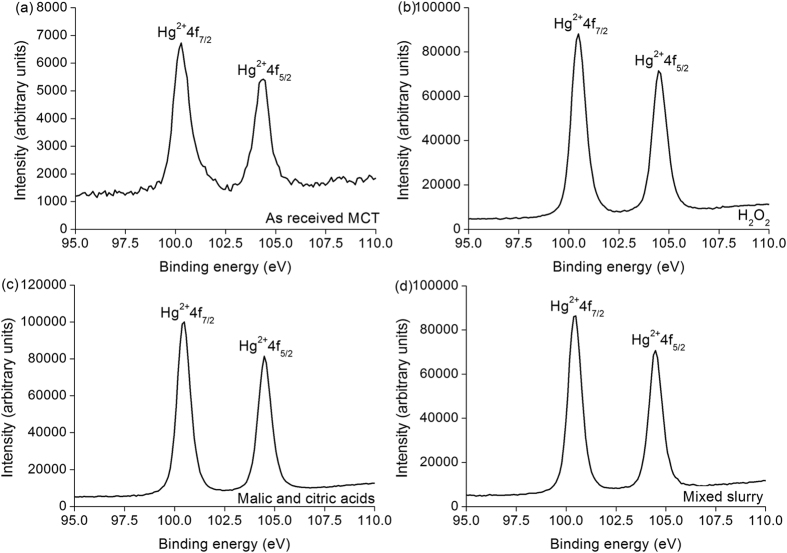
XPS spectra of Hg elemental valence states on (**a**) as-received, and (**b**) H_2_O_2_, (**c**) malic and citric acids, and (**d**) mixed slurry polished MCT wafers.

Figure 7 shows the XPS spectra of Hg elemental valence states on as-received, and H_2_O_2_, malic and citric acids, and mixed slurry polished MCT wafers. Hg^2+^4f peaks are present on all the MCT wafers^20^,^21^,^22^.

**Figure 8:**
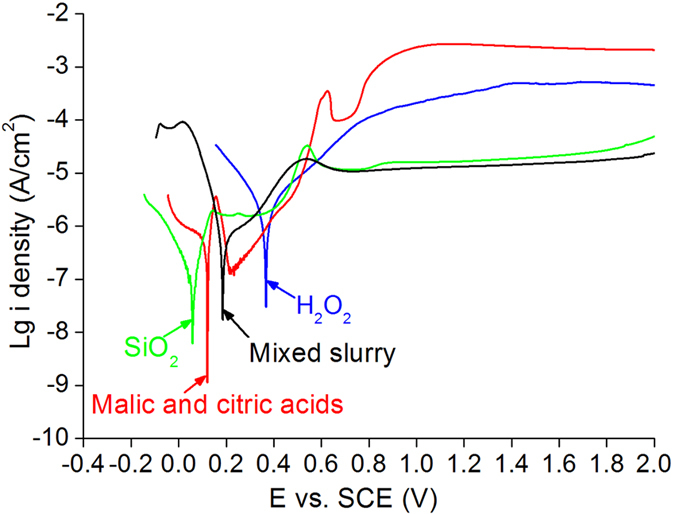
Electrochemical curves of current density as a function of potential versus SCE on MCT wafers polished by different slurries.

Figure 8 displays the electrochemical curves of current density as a function of potential versus SCE on MCT wafers polished by different slurry. Two passivated films are formed in each electrochemical curve. The corrosion potential of SiO_2_ is 0.06 V. The first passivated film of SiO_2_ is generated at 0.15 V, and stable at current density of 10^−5.8^ A cm^−2^ until 0.36 V. The second passivated film of SiO_2_ is produced at 0.68 V, and stable at current density of 10^−4.8 ^A cm^−2^ till 0.88 V. The corrosion potential of malic and citric acids shifts positively to 0.12 V. The first passivated film in malic and citric acids is formed at 0.16 V, and its current density is lower than that of SiO_2_ with increasing potential versus SCE from 0.18 to 0.4 V. The second passivated film is produced at 1.0 V, corresponding to the current density of 10^−2.6 ^A cm^−2^, basically stable and slightly lower with the increasing potential versus SCE. This is two orders magnitude higher than 10^−4.8 ^A cm^−2^ of SiO_2_. The corrosion potential of H_2_O_2_ is the highest at 0.36 V. However, there is no obvious transformation zone between the first and second passivated films of H_2_O_2_. The current density of H_2_O_2_ is 10^−3.7^ at 1.0 V, which is in the middle between those of SiO_2_ and malic and citric acids. The corrosion potential of mixed slurry is 0.18 V, consisting of H_2_O_2_, SiO_2_, and malic and citric acids, which is higher than those of SiO_2_ and malic and citric acids slurry. The current density of the first passivated film in mixed slurry is lower than that in SiO_2_, whereas the second passivated film of the former is approximately the same current density that of the latter. The malic and citric acids and mixed slurry have the lowest current density in the first and second passivated films among four solutions, respectively, meaning the malic and citric acids and mixed slurry dominating the first and second passivating process, correspondingly.

## Discussion

As MCT has soft and brittle nature, free abrasives of alumina are easy to embedding on the MCT wafers, as shown in Fig. 1. After embedding, it is difficult to remove the abrasives in the successive processes, resulting in the deteriorating of surface roughness. This is employed in the conventional machining processes for MCT semiconductors.

To avoid the embedding of free abrasives, fixed-abrasive lapping is used to machine the MCT wafers. Neither embedded abrasives nor cracks are found on the MCT wafers, except for uniform and subtle scratches, as observed in Fig. 2(a,b). The hardness of MCT is only 0.5 GPa^9^,^10^, which is much softer than 17 GPa of alumina^23^. Thus, ultrafine abrasives of alumina are used during lapping, obtaining lower surface roughness and saving time and cost for following CMP process. If the volume concentration of H_2_O_2_ is not appropriate, subtle scratches are left after CMP. Hereby, H_2_O_2_ plays an important role in removing the scratches, as illustrated in Fig. 2(c,d).

The diameters of SiO_2_ nanospheres distribute mainly at 20, 40, 60, and 120 nm, as observed in Fig. 3, leading to synergistically removing the passivated films formed in CMP. At an optimal volume concentration of ingredients, surface roughness R_a_, rms and PV values of 0.45, 0.55, and 4.74 nm are achieved, respectively with a measurement area of 70 × 50 μm^2^, as shown in Fig. 4(b). Thereby, the developed novel approach of CMP is extremely effective, to achieving mirror-like surface for MCT semiconductors with ultralow surface roughness.

The developed novel CMP slurry mainly consists of SiO_2_, H_2_O_2_ and malic and citric acids. SiO_2_ is commonly found in nature as quartz, and it is the major constituent of sand. Accordingly, SiO_2_ is environment-friendly. H_2_O_2_ solution is diluted by water, and has a volume percentage of 30%. H_2_O_2_ solution decomposes into oxygen gas and water in air slowly, and it is environment-friendly. In the developed CMP slurry, H_2_O_2_ solution is further diluted by SiO_2_ slurry and malic and citric acids. A finger dips in the mixed slurry containing H_2_O_2_ solution, and it turns light yellow. After flushing by tap water, the light yellow fades and the finger recovers the pristine color. Malic and citric acids are drinks, and popular in food industry. Consequently, malic and citric acids are environment-friendly. Accordingly, the developed CMP slurry is environment-friendly. Fixed-abrasive lapping uses the waterproof alumina papers as lapping pads, and the lapping solution is deionized water. After the CMP, deionized water and compressed air are used to clean and dry the MCT wafers, respectively. During CMP, neither strong corrosive acids and alkali nor toxic chemical regents are used, and the novel approach of CMP is environment-friendly.

Combining Figs 2(c) and 8, H_2_O_2_ and SiO_2_ dominate the forming of second passivated film, and the most scratches are removed, leaving subtle scratches on polished MCT wafers. SiO_2_ slurry is composed of nanospheres, as shown in Fig. 3. To disperse the nanospheres, OH^−^ is added into the SiO_2_ solution, resulting in the alkaline characteristic of SiO_2_ solution with pH value of 8.43. Wherefore, following equations are proposed^24^,^25^.


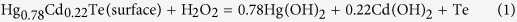



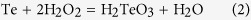



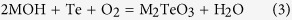



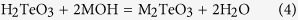



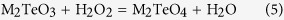



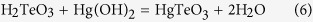



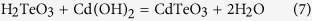



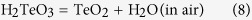


In equations (3), (4) and (5), the M represents metal ions in SiO_2_ slurry. Because H_2_O_2_ dissolves MCT well, most scratches are removed after CMP with low volume concentration of H_2_O_2_, as observed in Fig. 2(c).

MCT contains the Hg element, and it is easy to oxidize.


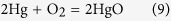



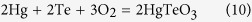



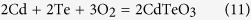


If MCT wafers are exposed in air for a bit long time, HgTeO_3_ and CdTeO_3_ are present^13^. For a short time in air, the reaction of equation (9) prevails, and therefore Hg^2+^4f are found prior to and after CMP on MCT wafers. Equation (11) needs a bit more time to complete, and only Cd^0^3d appears after removing the oxidized film of CdTeO_3_ on MCT wafers using H_2_O_2_, malic and citric acids and mixed slurry respectively, as shown in Fig. 6. In MCT, the elemental concentration of Hg is more than Cd, and equation (10) dominates the oxidizing process. Consequently, HgTeO_3_ are prominent in the oxidized film. During the first passivation process, malic and citric acids have the lowest current density, as shown in Fig. 8, indicating the less HgTeO_3_ left induced by malic and citric acids than by H_2_O_2_ solution. This results in the weaker Te^4+^3d peaks of malic and citric acids than those of H_2_O_2_ solution, as illustrated in Fig. 5(c,b). The mixed slurry has comprehensive advantages integrating the first and second passivated processes, compared to those in single H_2_O_2_ solution and malic and citric acids. Thus, the HgTeO_3_ are dissolved completely in the mixed slurry, Te^0^3d are present after CMP, as revealed by Fig. 5(d). During the first passivation process, following equations are presented. For SiO_2_ slurry includes OH^−^ ions^24^,^25^,


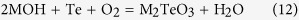


For malic and citric acids contain H^+^ ions,


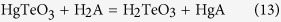



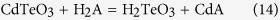



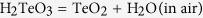
 (equation 8)


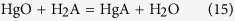


In equation (13), (14) and (16), A denotes an acid in malic and citric acids. For H_2_O_2_ solution,

Te + 2H_2_O_2_ = H_2_TeO_3_ + H_2_O (equation 2)


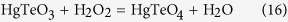



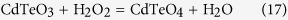



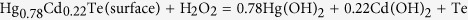
 (equation 1);


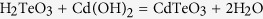
 (equation 6);


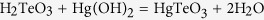
 (equation 7);


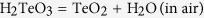
 (equation 8).

During the first passivation process in SiO_2_ slurry, only equation (12) takes place, resulting in its stable passivated current density, as shown in Fig. 8. For malic and citric acids, equations (13), (14), (15) and (8) happen simultaneously, the passivated current density varies sharply. For H_2_O_2_ solution, equations (1), (2), (6), (7), and (8) occur in both the first and second passivated processes, and therefore there is no obvious transformation zone between the first and second passivated films, as indicated in Fig. 8. The pH value of the environment-friendly CMP slurry varies from 4 to 5, which is adjusted by malic and citric acids, due to the dominating effect of malic and citric acids in the first passivation process. This is to achieve a good passivated effect using the environment-friendly CMP slurry, in terms of the extremely weak passivating effect of malic and citric acids in the second passivation process, as illustrated in Fig. 8.

In summary, a novel approach of CMP is proposed for MCT semiconductors. The whole machining process consists of lapping, CMP and cleaning, which are environment-friendly. This is different from conventional machining processes, in which free abrasive lapping and polishing are usually used, followed by chemical etching using bromine methanol. Bromine methanol is toxic to both the environment and operators, which is necessary to be replaced by environment-friendly slurry. In electrochemical measurements, the first and second passivation processes are confirmed. The first passivating process is related to the dissolution of oxidized films formed on the MCT wafers. The second passivating process is to dissolving the MCT wafers. During the first process, malic and citric acids plays a leading role, and the mixed slurry dominates the second process. The fundamental mechanisms of passivating processes are investigated using XPS and electrochemical measurements. Chemical reaction equations are proposed to elucidate the nature of developed novel approach of CMP for MCT semiconductors.

## Methods

### Specimens and conventional polishing

As-received Hg_0.22_Cd_0.78_Te (111) wafers were grown using modified Bridgman method, and used as specimens^10^. The wafers were 10 mm in diameter, and 0.8 mm in thickness. Both approaches were employed to machine the MCT wafers: one is the conventional polishing, and another is the novel approach of CMP. In conventional polishing, free abrasives of alumina were used with size of 1 μm, and the polishing pad was the nubuck leather. Three MCT wafers were distributed on the periphery of a circular plate of aluminium alloy in diameter of 150 mm. During conventional polishing, both the rotation rates of polishing pads and MCT wafers were 40 rpm. The pressure and polishing time on the MCT wafers were 15 kPa and 10 min, respectively.

### Novel approach of CMP using environment-friendly slurry

Fixed-abrasive waterproof papers of alumina were employed to lap the MCT wafers. The lapping slurry was deionized water. The waterproof papers were glued on a stainless steel plate, and used as the lapping pads. The MCT wafers were lapped by abrasive papers with sizes in a sequence of 5, 2, 1 μm. The lapping slurry was deionized water. The lapping pressure and time were 15 kPa and 10 s. The rotation rates of both MCT wafers and lapping pads were 40 rpm. After lapping, the MCT wafers were flushed and dried by deionized water and compressed air, respectively. The lapping pads were replaced by polishing pads of nubuck leather. Hydrogen peroxide (H_2_O_2_) was used as the oxidant with volume percentage of 30%. The pH adjustor was citric and malic acids. SiO_2_ slurry had a weight percentage of 50%. The polishing slurry of CMP mainly consisted of H_2_O_2_, SiO_2_ and citric and malic acids. The pressure and time of CMP were 22 kPa and 20 min, respectively. During the CMP, the rotation rates of both MCT wafers and polishing pads were 60 rpm. The optimal volume ratio of SiO_2_, H_2_O_2_ to malic and citric acids was 10: 4: 5. The optimal pH value of the CMP slurry varied from 4 to 5 adjusted by malic and citric acids. After CMP, the MCT wafers were cleaned and dried using deionized water and compressed air, respectively.

### Characterization

Surface morphology was characterized using an optical microscope (Olympus MX40, Japan). Surface roughness was measured by a precision non-contact surface profilometer (Zygo, NewView 5022, USA). SiO_2_ nanospheres were characterized using a transmission electron microscope (Tecnai spirit, FEI, Netherlands). Electrochemical measurements were conducted by an advanced electrochemical system (PARSTAT 2273, Princeton Applied Research, Ametek, Inc.). The pH values were 2.95, 4.56, 8.43, 7.54 in electrochemical measurements for H_2_O_2_, malic and citric acids, SiO_2_, and mixed slurry consisting of H_2_O_2_, SiO_2_ and malic and citric acids, respectively. Saturated calomel electrode (SCE) of potassium chloride (KCl) and platinum with purity of 99.99% were used for referenced and auxiliary electrodes, respectively. XPS spectra were measured using a VG ESCALAB MKII spectrometer with a magnesium Kα excitation source.

## Additional Information

**How to cite this article**: Zhang, Z. *et al.* A novel approach of chemical mechanical polishing using environment-friendly slurry for mercury cadmium telluride semiconductors. *Sci. Rep.* **6**, 22466; doi: 10.1038/srep22466 (2016).
